# Grasp-squeeze adaptation to changes in object compliance leads to dynamic beta-band communication between primary somatosensory and motor cortices

**DOI:** 10.1038/s41598-022-10871-z

**Published:** 2022-04-26

**Authors:** Huy Cu, Laurie Lynch, Kevin Huang, Wilson Truccolo, Arto Nurmikko

**Affiliations:** 1 School of Engineering, Brown University, Providence, RI USA; 2 Department of Neuroscience, Brown University, Providence, RI USA; 3 Carney Institute for Brain Science, Brown University, Providence, RI USA; 4 Department of Neurosurgery, Brigham and Women’s Hospital, Harvard Medical School, Boston, MA USA

**Keywords:** Sensorimotor processing, Computational biology and bioinformatics

## Abstract

In asking the question of how the brain adapts to changes in the softness of manipulated objects, we studied dynamic communication between the primary sensory and motor cortical areas when nonhuman primates grasp and squeeze an elastically deformable manipulandum to attain an instructed force level. We focused on local field potentials recorded from S1 and M1 via intracortical microelectrode arrays. We computed nonparametric spectral Granger Causality to assess directed cortico-cortical interactions between these two areas. We demonstrate that the time-causal relationship between M1 and S1 is bidirectional in the beta-band (15–30 Hz) and that this interareal communication develops dynamically as the subjects adjust the force of hand squeeze to reach the target level. In particular, the directed interaction is strongest when subjects are focused on maintaining the instructed force of hand squeeze in a steady state for several seconds. When the manipulandum’s compliance is abruptly changed, beta-band interareal communication is interrupted for a short period (~ 1 s) and then is re-established once the subject has reached a new steady state. These results suggest that transient beta oscillations can provide a communication subspace for dynamic cortico-cortical S1–M1 interactions during maintenance of steady sensorimotor states.

## Introduction

Task-specific sensorimotor control is crucial for primates to perform complex hand movements for dexterous manipulation of objects. The question we asked in this work is how key cortical areas contribute to our ability to squeeze a *compliant* object with a measured amount of grip force by focusing on the interplay between the primary motor cortex (M1) and the primary somatosensory cortex (S1). It is known that in generating outputs for dexterous manipulation^1^ or participating in motor skill learning^2^, M1 plays an essential role. The primary somatosensory cortex (S1) is known to be crucial in receiving tactile sensory input^3^. Among studies of how tactile stimuli are encoded in S1, Weber et al.^4^ studied how spatial and temporal codes mediate tactile perception of static natural textures while Chowdhury et at^5^ showed how its Area 2 also encodes proprioception in the whole arm kinematics. Romo and Rossi-Pool^6^ published a broad review of the role of S1 to encode a sense of active touch in macaques. More generally, Jazayeri and Movshon^7^ searched for optimal representation of sensory information from neural population dynamics. Importantly, in terms of potential applications, understanding the dynamical interplay between motor and somatosensory cortices can be of relevance in ongoing efforts to advance brain-machine interface to include sensorimotor functions^8,9^.

Descriptions of cortico-cortical communication include ideas about oscillatory transients and related wave propagation^10–15^. In particular, the emergence of transient beta oscillations in sensorimotor cortex during movement preparation periods, their attenuation during movement execution, as well as related directed interactions between sensorimotor areas in this frequency band have been examined previously^12^. On the other hand, it is also well known^16^ that the emergence of transient beta oscillations is a hallmark during the execution of (isometric force) precision-grip tasks, where M1 is active in maintaining states of little or no changes in the corresponding kinematics.

Against this backdrop, we asked the question of the role of beta oscillations in facilitating cortico-cortical communication between S1 and M1, specifically when subjects squeezed an elastically deformable object to attain a specific instructed force level. We implanted intracortical microelectrode arrays (MEA) in the hand area of both S1 and M1 in two macaques and simultaneously recorded the joint neural population dynamics during a grasp-squeeze-and-hold task. In the first sets of experiments, labeled as the “NORMAL” task the animals had to apply a specific force for several seconds in squeezing the compliant manipulandum in steady state. In the second set of experiments, the compliance of the target object was changed abruptly (perturbation), unbeknownst to the animal. This is the task we label as the “SURPRISE EVENT”. The animals now had to find another steady state by modifying the strength of their grasp in real time. To our knowledge, our study is the first to address directed neural dynamics of cortico-cortical interactions between S1 and M1 when dexterous manipulation involves deformable objects. In contrast to previous studies, it also involves two main steady states separated by a perturbation. It is expected that these tasks require that a subject engages sensory feedback via S1 to correctly update M1’s motor outputs.

We recorded both spikes and local field potentials (LFP) across the two MEAs but focus on the explicit role of LFPs in this paper, specifically the role of transient beta oscillations in reflecting communication between S1 and M1. Directional influences within the sensorimotor network involving beta oscillations were reported by Brovelli et al.^12^ and analyzed using Granger Causality (GC). Likewise, we use in this paper a nonparametric version^17^ of the GC metric^18,19^ as the main statistical approach to study the direction and strength of the S1 ↔ M1 communication. Statistical assays were applied to analyze the multichannel data. We show below that cortico-cortical communication between S1 and M1 is dynamic, varying across the various stages of the reach-grasp-squeeze task of a compliant object. The directed interactions are frequency-dependent and strongest when the animal is successfully squeezing the object by maintaining the correct force during a steady-state lasting several seconds. However, when an abrupt change in the compliance is imposed externally, we observed that the S1 ↔ M1 communication is modulated dynamically in a manner which may suggest an important role of S1-M1 circuits while a new steady state is reached.

## Methods

### Ethics statement

All research protocols were approved and monitored by Brown University Institutional Animal Care and Use Committee, and all research was performed in accordance with relevant NIH guidelines and regulation. The manuscript followed the recommendations in the ARRIVE guidelines.

### Animal preparation

Two male rhesus macaque monkeys (identified as Monkey M and Monkey C, respectively: body weight, 9.5–13.0 kg, age, 6–11 years old) were trained to sit in a custom-made chair to restrain head and body movements. The chair was placed next to the experimental apparatus on a table with height set at the waist level of the monkey to simplify the arm and hand kinematics for reach, grasp, and rest.

### Behavioral apparatus

**Figure 1:**
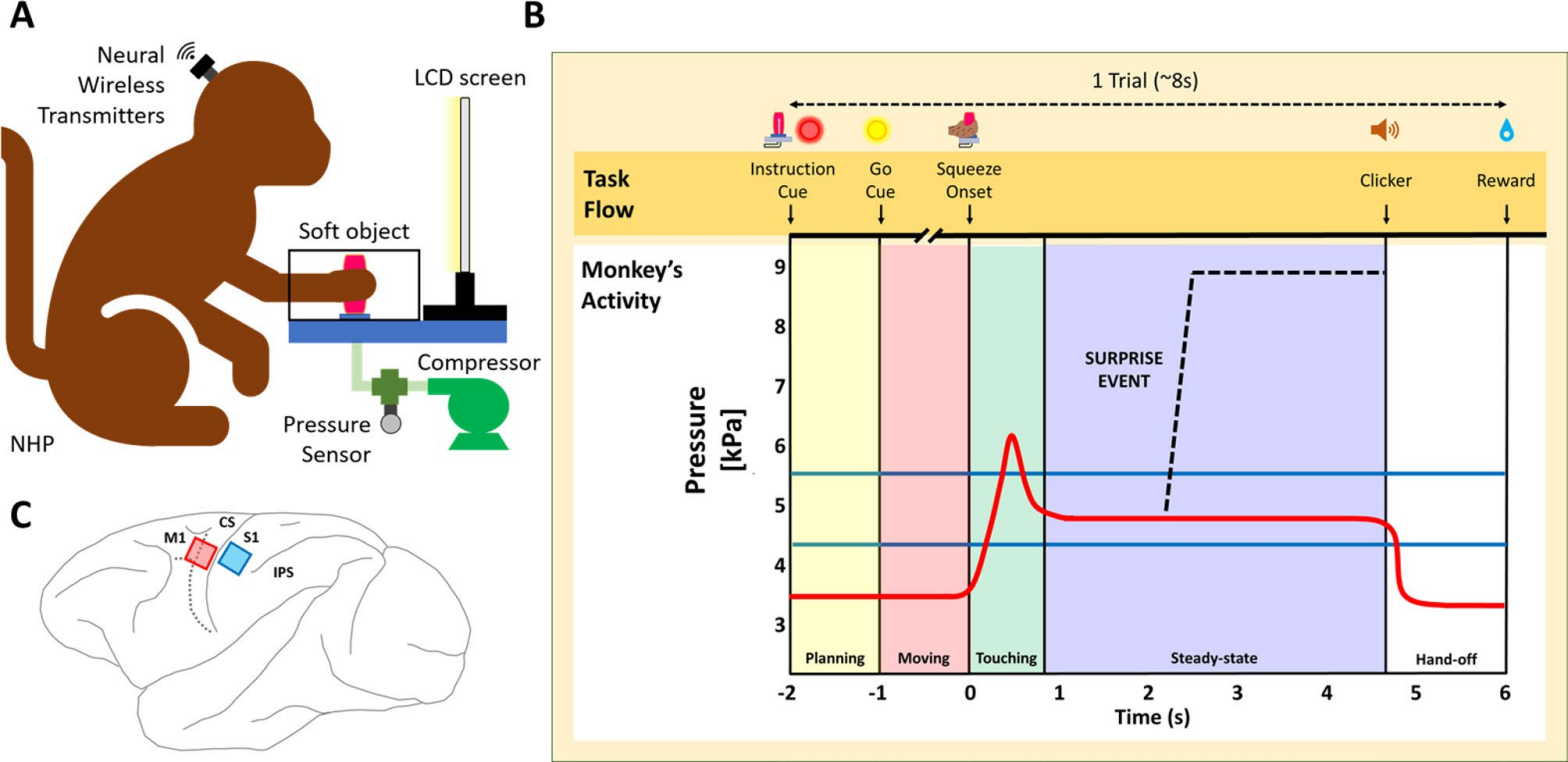
Experimental design. (**A**) Schematic of the experiment. Central to the design is a compliant cylindrical manipulandum composed of a soft polymer envelope which is internally pressurized from a hydraulic air compressor. The monkey sits in a custom-made chair and reaches for the manipulandum through an opening in a transparent window. Multichannel signals are collected simultaneously from a pair of microelectrode arrays (MEAs in S1 and M1) and transmitted wirelessly to nearby neural signal processing electronics. (**B**) Time course of the task. Top: Graphical representation of task flow. The task flow is separated into four stages: Movement Planning (Yellow rectangular panel), Movement, i.e. arm/hand reach (Pink panel), Touch and Grasp (Green panel), and Steady State squeeze (Blue panel). The solid red line represents trial averaged time varying pressure inside the manipulandum as the monkey first grasps and then squeezes the manipulandum to reach a steady-state. In addition to this ‘NORMAL’ task, the dashed black line shows schematically an abrupt elevation in the internal pneumatic pressure of the manipulandum introduced during the steady state, labeled as the ‘SURPRISE EVENT’ the onset of which was randomly timed from 2–5 s after the onset of Touch. The two blue horizontal lines define the target pressure range within which the monkey must maintain the force of hand squeeze in steady state to complete a successful trial. (**C**) Schematic of the approximate cortical location of the implanted intracortical MEAs. *CS* central sulcus, *IPS* intraparietal sulcus, *M1* primary motor cortex, *S1* primary somatosensory cortex.

The focus of the experimental apparatus was a compliant object for monkeys to squeeze by using their preferred hand: a pressurized, hollow cylindrical rubber manipulandum (4 cm in diameter, 12 cm in height, and 1 mm in thickness) molded from bulk silicone elastomer (Fig. 1A). An aperture at the bottom of the rubber manipulandum was connected to a pneumatic system to determine its internal pressure. The surface roughness of the manipulandum prevented any slippage of the monkey’s hand. The manipulandum was housed inside a target chamber made of acrylic with dimensions of 25 × 25 × 12.5 cm. A circular opening was cut into the acrylic to allow the monkey’s arm and hand to reach through to the manipulandum while constraining the range of reach. The setup included an LED for visual cue to start the task, a liquid reward system, and an LCD screen giving the animal visual feedback about the force of squeeze. The setup was equipped with a video camera tracking system to observe the arm/hand kinematics to help assess the monkey’s performance and to time the onset of its touch and grasp of the manipulandum. The compliance of the manipulandum was set by controlling its internal hydrostatic pressure from a gas manifold (Fig. 1B). The system consisted of an air compressor (#FP209499AV, Campbell Hausfeld) and two-way 24 VDC miniature valves (#411L1124HV, ASCO) powered by a common 24 VDC power source and controlled through an Arduino and NI DAQ board. The internal pressure was recorded using a pressure sensor (#MPX5050GP1, NXP USA). The entire experiment was controlled by a custom-made virtual instrument interface in LabVIEW (National Instruments, Austin, TX, USA).

### Behavioral task

We trained the two non-human primates (NHP, *Macaca mulatta*) on a grasp-squeeze-and-hold task of the soft manipulandum. The animals’ heads were loosely restrained during the task to prevent up-down and side-to-side head movements. The animals were trained to first place their hands to on a pad adjacent to the manipulandum during the preparatory resting phase, then to wait for the LED cue to initiate the reach and grasp (Fig. 1A). The LCD screen behind the manipulandum displayed a thick horizontal black stripe against white background and a vertically moving red indicator dot. The vertical position of the dot informed the level of pressure inside the manipulandum while the black stripe represented the desired pressure induced by the animal’s squeeze. The animals had to position the red dot entirely within the width of the black stripe to gain a reward. The minimum acceptable pressure/force was 4.5 kilopascals (kPa), equivalent to 11.25 Newton for a 25 cm^2^ estimated hand area while the largest acceptable pressure was 5.5 kPa (blue horizontal lines in Fig. 1B). The total internal pressure is the sum of the pneumatically set base value plus the added pressure applied by the monkey. After reaching the target pressure, each animal was required to maintain a constant and steady force for at least 4 s before retrieving its hand (plateau region in the pressure vs. time trace in red in Fig. 1B). Following a good trial, the experimenter turned off the cue LED and proffered a clicker sound to signal the monkey to release his hand from the manipulandum and place it back to the resting position. The monkey had to maintain the resting position for at least 1 s for the juice reward to start. The intertrial interval was 2–4 s. A trial was considered successful if the monkey could complete all the steps. The monkeys were able to learn and adapt to the task quickly after a few weeks of training and were considered trained in this ‘NORMAL’ task if their performance was above 80% for 5 consecutive days.

Once the monkeys were routinely performing the NORMAL task, we introduced an abrupt confound, unbeknownst to the animals, in a series of random trials. The confound was a sudden reduction in the compliance of the manipulandum during the steady squeeze (dashed line in Fig. 1B) induced by increasing the level of the internal pressure in the manipulandum by approximately 4 kPa from its baseline value within ~ 200 ms and without any visual or auditory cue to the animal. Following this “SURPRISE EVENT”, the monkeys had to re-adjust the force of their squeeze to adapt to the new state of compliance so as to reach and maintain a new steady squeeze for up to another five seconds (guided by the LCD pressure indicator). The new acceptable range was 8–10 kPa, allowing for variability in the excess pressure applied to the manipulandum. The thick horizontal black stripe and the red indicator ball displayed on the LCD screen were shifted to new positions, accordingly.

### Intracortical microelectrode arrays

Following the training, each monkey was implanted with two 100-element microelectrode arrays (MEA, Blackrock Microsystems, Salt Lake City, UT, USA) targeting the hand representation of the primary somatosensory (S1) and the primary motor (M1) cortices, respectively (Fig. 1C). To locate the hand areas, we reconstructed a 3D model of the monkey’s head fusing their MRI and CT scan images. We combined the data while using the Macaque brain atlas and previous studies^20,21^ to locate the implant sites. A large craniotomy (30 × 40 mm) was performed over the motor and somatosensory cortices. For both monkeys, the 4 × 4 mm^2^ arrays were implanted about 1 mm away from and symmetrical to the central sulcus. We first located the central sulcus (CS) and intraparietal sulcus (IPS). The S1 array was implanted next to the tip of the IPS and the M1 array was located symmetrically to the CS. We sutured the dura and attached the bone flap using 3 titanium strips (Stryker). Each MEA consists of 96 electrodes with 400 um pitch, targeting layers IV/V and V of S1 (1-mm-long electrodes) and M1(1.5-mm-long), respectively. The monkeys were returned to the experiments following a recovery period of 2 weeks.

### Electrophysiology instrumentation

Neural signals from each array were recorded, amplified (5000 ×), anti-alias analog bandpass filtered from 0.3 Hz to 7.5 kHz, digitized (16-bit Analog-to-digital converter, 12-bit transmission), and simultaneously sampled at a rate of 30,000 Samples per Second. The neural signals were transmitted by a Cereplex W wireless headstage (Blackrock Microsystems, Salt Lake City, UT) in the 3.2–3.8 GHz bands to two RF antennas. Antennas were placed near each other, within 1 m from the transmitter and oriented 90° from each other. Data were acquired by two CerePlex W receivers and processed by two Cerebus Neural Signal Processor (NSP). Other experimental data, including the transient pressure and timing signals of the LED cue and camera systems, were fed into the Blackrock workstation sampled at 30,000 Samples per Second. The digital signals were used to align data from the S1 and M1 arrays temporally. The data was stored in a workstation and analyzed using MATLAB software (Mathworks Inc., Natick, MA, Version 2019a).

### Preprocessing the data

The instantaneous internal pressure in the manipulandum was used to time stamp the moment when the animal touched the manipulandum (“Touching” in Fig. 1B) in the NORMAL task as well as the instance of the abrupt increase of compliance in “SURPRISE EVENT” task. Using the digital synchronizing signal from cue/pressure readout electronics, we timed the behavioral dynamics with the transient pressure within the manipulandum. The baseline pressure value at the onset of each trial was computed by averaging the pressure value during a 1-s period prior to the monkey’s movement planning phase. We defined the onset of “Touching” when the pressure had crossed 5% of the baseline value. The onset of the SURPRISE EVENT was defined in the same manner. For each trial, we aggregated the behavioral and neural data from all recorded channels to inspect the quality of the recorded data. All obviously bad trials were removed prior to further analysis.

As noted, we focused in this paper on the extraction of field potentials (LFPs). First, we removed the 60-Hz noise from the raw data using a second-order IIR notch filter built by MATLAB ‘iirnotch’ and ‘filtfilt’ functions. Trials or channels corrupted by 60-Hz interference were removed before further analysis. Then, we lowpass filtered the signals using a Butterworth filter with the lowpass frequency of 100 Hz built by MATLAB ‘butter’ and ‘filtfilt’ functions. Signals were downsampled to 400 S/s using ‘downsample’ functions. We also removed trials from the pool that had abnormally large LFP amplitudes.

To estimate average Event-Related Potentials (ERPs) and their 95% confidence regions, we used bootstrap resampling method. For each animal’s data, we sampled with replacement of all trials collected from three sessions of LFP data and took the average to create a resampled ERP. In subject M, there were 51 trials in the three sessions, respectively; in subject C there were 86 trials in the three sessions. We repeated this resampling step 1000 times to acquire the ERP distribution. For each time point during the task, we computed the 2.5% and 97.5% percentiles to reach the 95% confidence level.

### Granger causality and coherence

To characterize the functional link between S1 and M1, we computed the nonparametric bivariate Granger causality between LFP time series from pairs of M1 and S1 channels in both the time and frequency domains. Granger causality^17,18,22^ is a statistical measure of causality that is well-suited to define the bidirectional interactions between multiple brain areas and information flow from raw data. There have been many applications of Granger causality analysis to study interareal interaction in the cortex^23^. Here, we utilized a nonparametric approach^17^ to compute pairwise Granger Causality between each S1 and M1 LFP signal pairs. First, we estimated the spectral density function from raw LFP data using the multitaper approach. In our analysis, we used 7 tapers to estimate the spectral density matrix. Spectrograms and coherence were derived directly from the matrix of auto- and cross-spectra. We used the algorithms provided in^22^ to implement Wilson’s algorithm for spectral matrix factorization of the matrix and computed the bivariate Granger causality by using Geweke’s formula for Granger causality. To evaluate the statistical significance of Granger Causality within our data, we ran the random permutation test over 200 permutations. To do so, for each iteration and each LFP pair, we permuted the order of trials and computed the Granger causality. Finally, we computed the p-value counted from the times when randomized GC are larger than the real GC from the following formula:

$$p\_values= \frac{\left(1 + \#\left\{G{C}_{rand}> G{C}_{true}\right\}\right)}{1 + \# \, of\, permutations}$$

Given the two 96-element intracortical arrays, we have 9,216 pairs. We applied multiple testing corrections^24^ with False Discovery Rate (FDR) to find the corrected p-value and then selected significant pairs based on those corrected p-values for a chosen target α = 0.05.

## Results

For each animal, we recorded 3 consecutive training sessions and concatenated those datasets to get the finals with 51 trials from monkey M and 86 trials from monkey M. All trials for analysis were filtered (“Methods”). For the ‘NORMAL’ task, we aligned the data to the onset of touch and truncated the data from −2 s to 3 s marks to get 5 s long segments. For the ‘SURPRISE EVENT’ task, we aligned the data to the onset of the compliance change and truncated the data from -2 s to 3 s marks. Out of the total of 9216 possible LFP channel pairs, we selected 1310 pairs in monkey M and 898 pairs in monkey C which showed significant causality across the multiple stages of Fig. 1B. We chose a target α = 0.05 and used FDR to correct for multiple testing (Methods). We only claimed statistically significant values for a target α = 0.05, with FDR correction for multiple testing. The Causality index GC was evaluated for each direction of cortical communication, i.e., computed GC separately for S1 → M1 and M1→ S1, respectively.

### Beta oscillations in S1 and M1 during the ‘NORMAL’ task

**Figure 2:**
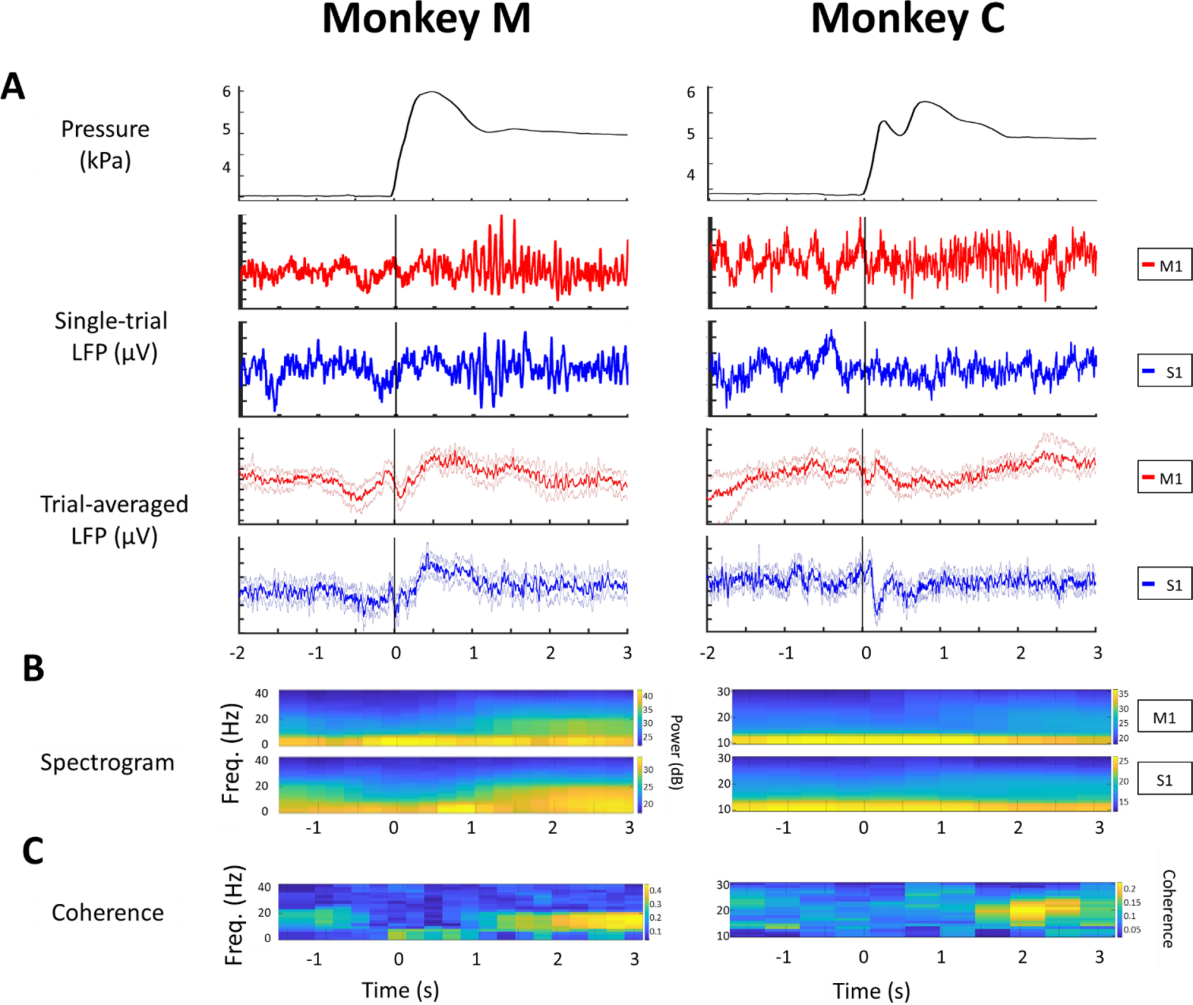
Single LFP S1-M1 channel pair activity in the ‘NORMAL’ reach-and-grasp task. All trials were aligned to the onset of touch (t = 0). Left: Monkey M. Right: Monkey C. (**A**) Upper panel: Example of the typical pressure profiles showing similar temporal force patterns applied by each animal: An initial overshoot from 0 to 1 s, followed by a stable isometric force (steady-state). Middle panels: LFP signals recorded from one M1 and one S1 channel showing the beta-band activity during steady-state. Lower panels: Trial-averaged LFP response on one channel for both S1 and M1 signals showed modulation at the onset of touch (bootstrap resampling, the number of resamples is 1000). The bold red and blue lines showed the mean of LFP amplitude across trials and the shaded regions denoted 95% confidence interval of the data (n = 51 trials collected in 3 sessions for monkey M and n = 86 trials collected in 3 sessions for monkey C). (**B**) Trial-averaged LFP spectrogram during the ‘NORMAL’ task showing downmodulation at the onset of touch and increased beta activity in steady state. Upper panel: M1 spectrogram. Lower panel: S1 spectrogram. (**C**) Spectra-temporal S1–M1 LFP coherence shown as a heat map, highlighting the beta coherence (Random permutation test, #permutation = 200, p = 10^–2^) during the steady state. Note that the beta-band in Monkey M from 13 to 16 Hz, and monkey C from 23 to 30 Hz**.**

Both monkeys performed the trained task in a stereotypical sequence: Following initial movement planning and subsequent arm reach, the animals initially overshot the set target force after grasping on the manipulandum. A transient peak in the internal pressure of the manipulandum occurred within 0.2–0.4 s after the onset of touch (shown schematically as red solid line Fig. 1B, actual measurement for each monkey shown in upper trace of Fig. 2A). The monkeys then reduced the force of their squeeze to attain the correct pressure mark within approximately 1 s. As per the instructed task, they continued to hold and maintain the correct and constant force for several seconds in steady state.

Upon touching the manipulandum, a relatively slow transient modulation in the LFP amplitude and spectrum was recorded from both electrode arrays (Fig. 2A,B). Following the decay of these transients, well-defined oscillations commenced in each area, remaining pronounced through the steady-state, constant squeeze stage. In analyzing the multichannel data from pairs of MEA channels (one channel from S1, another from M1), we tracked the modulation of the LFPs in both amplitude (bootstrap resampling, the number of resamples is 1000) and frequency across each particular stage of the task (‘Planning’, ‘Moving’, ‘Touching’, and ‘Steady-state’, each defined by a colored rectangle in Fig. 1B). For the movement ‘Planning’ stage, we observed that LFP powers remained quite stable in most M1, S1 channels (160/192 channels in monkey M and 123/161 channels in monkey C). However, for the ‘Moving’ and ‘Touching’ stages, we documented a significant decrease in the amplitude of the LFP signals across all channels once the monkeys initiated the arm movement and opened the aperture of the hand to grasp the object. This down-modulation began some two hundred milliseconds before the actual onset of touch (Fig. 2A) with M1 appearing to precede that of S1 by a several milliseconds. Both modulations shared a similar evoked potential waveform, namely a bipolar waveform led by a faster negative transient followed by a slower positive repolarization. The LFP amplitudes then re-emerged within approximately 0.5 s after the onset of touch. We also observed similar downmodulation in the M1 and S1 LFP signals when the monkeys retrieved the hand upon completing a trial.

The dominant oscillatory features recorded from both S1 and M1 frequency matched the range of known beta-range oscillations (15–30 Hz, Fig. 2B). The beta oscillations were first evident in both S1 and M1 during the movement ‘Planning’ stage (−2 to −1 s). That beta oscillations decrease upon initiation of arm kinematics is of course well-known—here we see beta being replaced by the emergence of a transient low-frequency oscillation. These putative ERPs remained the dominant feature from movement initiation to approximately 1 s after onset of touch, i.e., while the monkeys applied too much force. Upon entry to the steady state (flat region in red solid line of Fig. 1B), the beta- oscillations re-emerged and attained dominance after t > 0 s, remaining well-defined to the end of the task. Quantitatively, the beta-band power in M1 was larger than in S1 during the steady-state squeeze in monkey M (40 $\frac{{\mu V}^{2}}{Hz}$ vs 33 $\frac{{\mu V}^{2}}{Hz}$**,** random permutation test, p-value = 5 × 10^–3^) as well as monkey C (25 $\frac{{\mu V}^{2}}{Hz}$ vs 19 $\frac{{\mu V}^{2}}{Hz}$**,** random permutation test, p-value = 10^–2^, Supplementary Fig. 1A). Figure 2C shows the coherence profile of S1 and M1 through the task. The analysis shows good consistency between the LFP spectrograms and computed coherence.

### Granger causality shows bi-directional communication between the two areas

**Figure 3:**
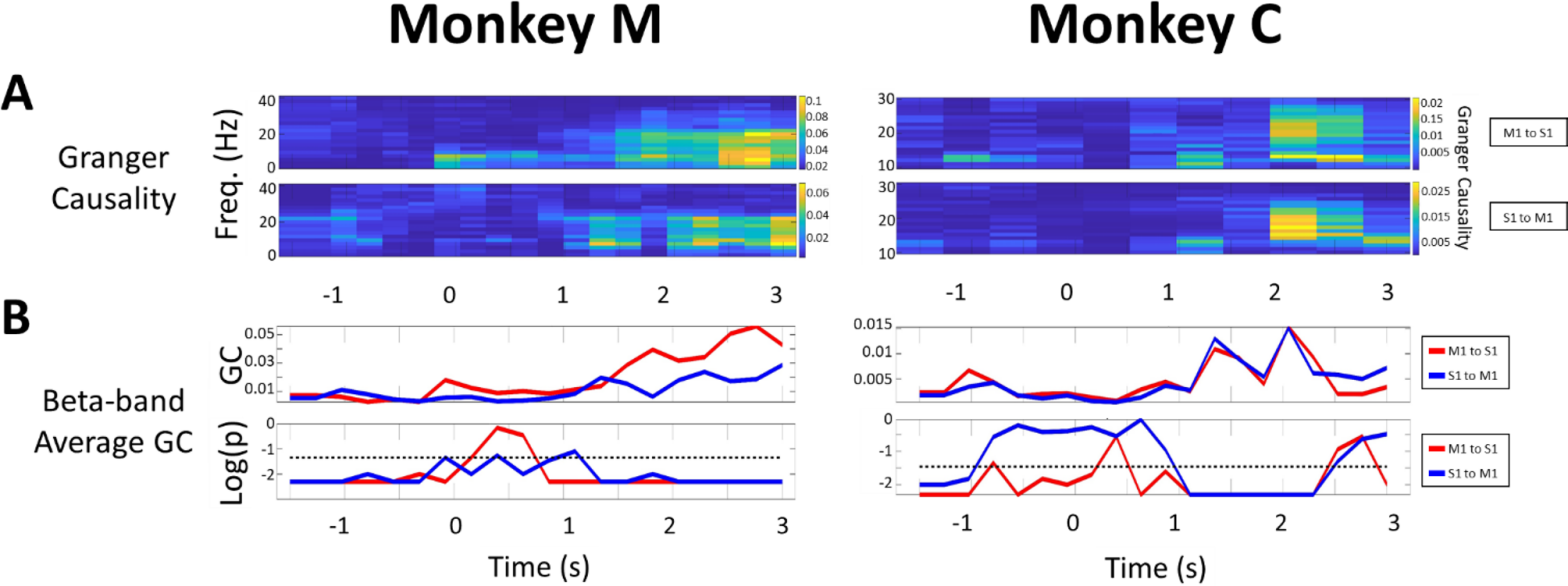
Example of Granger Causality of a single S1-M1 LFP channel pair revealed bidirectional communication between the two cortical areas during the ‘NORMAL’ task. All trials are aligned to the onset of touch (t = 0). Left: Monkey M. Right: Monkey C. Data for the selected channel pair was averaged across all trials (n = 51 for monkey M and n = 86 for monkey C). (**A**) Spectra-temporal S1–M1 LFP Granger causality (GC) heat map shows a bidirectional beta-band relationship between S1 and M1 for both monkeys. Causality increased significantly and reached its maximum during the steady-state. Upper panel: GC index for M1→ S1. Lower panel: GC index for S1→ M1. (**B**) Upper panel: Beta-band (15–30 Hz) averaged GC of a single LFP pair showed distinct dynamics and directionality across the four distinct stages of the task; Red trace for M1→ S1, Blue trace for S1→ M1). The GC indices are low in the ‘Planning’, Moving’, and ‘Touching’ stages but increase rapidly when the animal reached the steady-state hand squeeze. Lower panel: p-value on log_10_ scale calculated from a random permutation test (# permutations = 200). We chose a target α = 0.05 and used FDR correction for multiple testing. The black dashed line represents the log of adjusted α. Those GC indices with p-value above the dashed line were considered as statistically not significant.

Performing a nonparametric Granger causality test for selected single S1-M1 LFP pairs, we found that the GC index is bidirectionally modulated across the temporal pressure profile of the manipulandum (proportional to the force of the monkey’s squeeze). Figure 3A displays an example of spectro-temporal Granger causality as heat maps for the two animals showing the presence of bidirectional communication between S1 and M1 across the range of beta-band oscillations, approximately 13–16 Hz in Monkey M and 23–30 Hz in Monkey C. Age differences have been implicated in variation of the beta band frequency and power in healthy humans ^25,26^. Given that one of our monkeys was old and the other was just reaching adulthood, we suggest that the age factor can be a contributor to the differing beta frequencies.

In both animals, there were only modest S1–M1 detected interactions present during initial resting and movement planning stages (−2 to −1 s). These interactions decreased further while the monkey executed the movement of his arm and hand (−1 to 0 s) and remained low during the ‘touching’ stage (0 to 1 s). However, once a grasp was established, the GC index increased bidirectionally during the progression toward the steady-state. Consistent with the computed coherence, the GC values reached the maximum during steady-state in both monkeys. Figure 3B shows the time course of beta-band averaged GC during the steady-state and its directional dependence (red trace for “M1 → S1” in Fig. 3B ;“blue trace for S1 → M1” in Fig. 3B).

**Figure 4:**
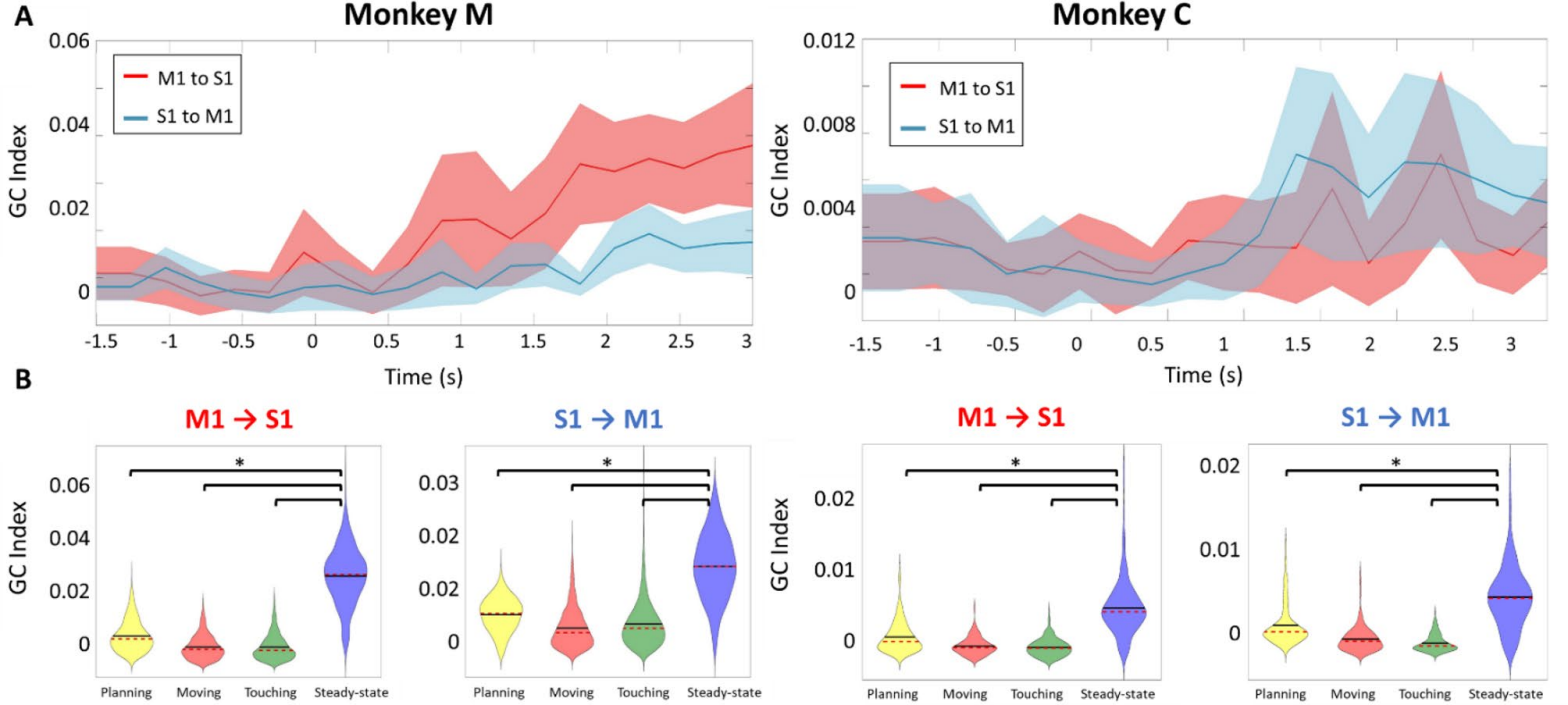
Channel-averaged Granger causality shows bidirectional S1–M1 communication during the steady-state hand squeeze. Left: Monkey M. Right: Monkey C. All trials were aligned to the onset of touch (t = 0). (**A**) Beta-band averaged, and channel-averaged Granger causality index computed using a sliding window (length = 500 ms, step = 250 ms) for each direction of putative cortical communication. The p-value was calculated from random permutation test (# permutations = 200). We chose a target α = 0.05 and used FDR correction for multiple testing. M1→ S1, Red shadowed trace; S1→ M1, Blue shadowed trace. The bold red and blue lines showed the mean of GC values across LFP pairs, and the shaded regions denoted one standard deviation of the data. (**B**) Violin plots of the distribution in the GC index from the pool of 1310 selected channel pairs in monkey M and 898 pairs in monkey C for the four stages of the task. The GC values during the Steady-state are confirmed to be the largest (*p = 10^–3^, two-sample t-test).

For a more comprehensive analysis of data in the ‘NORMAL’ task, we evaluated the average beta-band causality index across S1 and M1 channels pairs (1310 selected pairs in monkey M and 898 pairs in monkey C). Viewed across the entire time course of the trials, the causality analysis shows firstly that the statistically evaluated GC index was significant during the initial resting/planning stages when the monkey was resting his hand. The GC index then decreased slightly during the movement kinematics (arm and hand reach). Causality remained low and statistically insignificant during the ‘Touching’ stage. A robust bidirectional increase then followed reaching maximum once the animals entered the steady-state (Fig. 4A), consistent with the example result above for a single individual LFP channel pair.

To evaluate the distribution of the GC index across S1 and M1 channel pairs, we selected specific timepoints at midpoint of each of the four stages, at −1.5, −0.5, 0.5, 1.5 s marks, respectively. We implemented a kernel density estimation to compute the distribution of GC over selected pairs. Figure 4B shows the distribution of the GC index expressed as violin plots across different task stages. The GC values during steady-state squeeze were significantly larger than for other stages (two-sample t-test, p = 0.001). The violin plots lend support the hypothesis of dynamical modulation in the bidirectional S1 ↔ M1 communication during the squeeze of the compliant manipulandum.

To investigate if one cortical area was the principal driver in the inter-area relationship, we compared values of the GC index for S1→ M1 and M1→ S1, respectively, by selecting 10 channel pairs which had the GC index closest to the mean of the distribution and performed a random permutation test (1000 permutations) at each stage of the task for each pair (Supplementary Fig. 2A). We found that in the planning stage, both S1→ M1 and M1→ S1 communication is significant, sharing the same strength. During the movement stage, both communication strength decreased in value but continued to show bidirectionality (in causality). Entering the “touching” stage we find that in monkey M in particular, the GC index for M1→ S1 becomes larger. This particular direction of inter-areal communication (M1→ S1) further increases and takes on a dominant role once in the steady state (p = 0.001, random permutation test). However, in monkey C, we found results which were more ambiguous. Here, a majority, 7 of 10 channel pairs, shows that index for S1→ M1 is dominant in the steady-state while a minority, 2 of 10 pairs, show that the GC indices are comparable, and only 1 channel pair supports M1→ S1’s dominance (Supplementary Fig. 2B). This ambiguity between the animals will be discussed further in the “Discussion” section.

### An abrupt, random change in manipulandum compliance modulates LFPs in both amplitude and frequency

**Figure 5:**
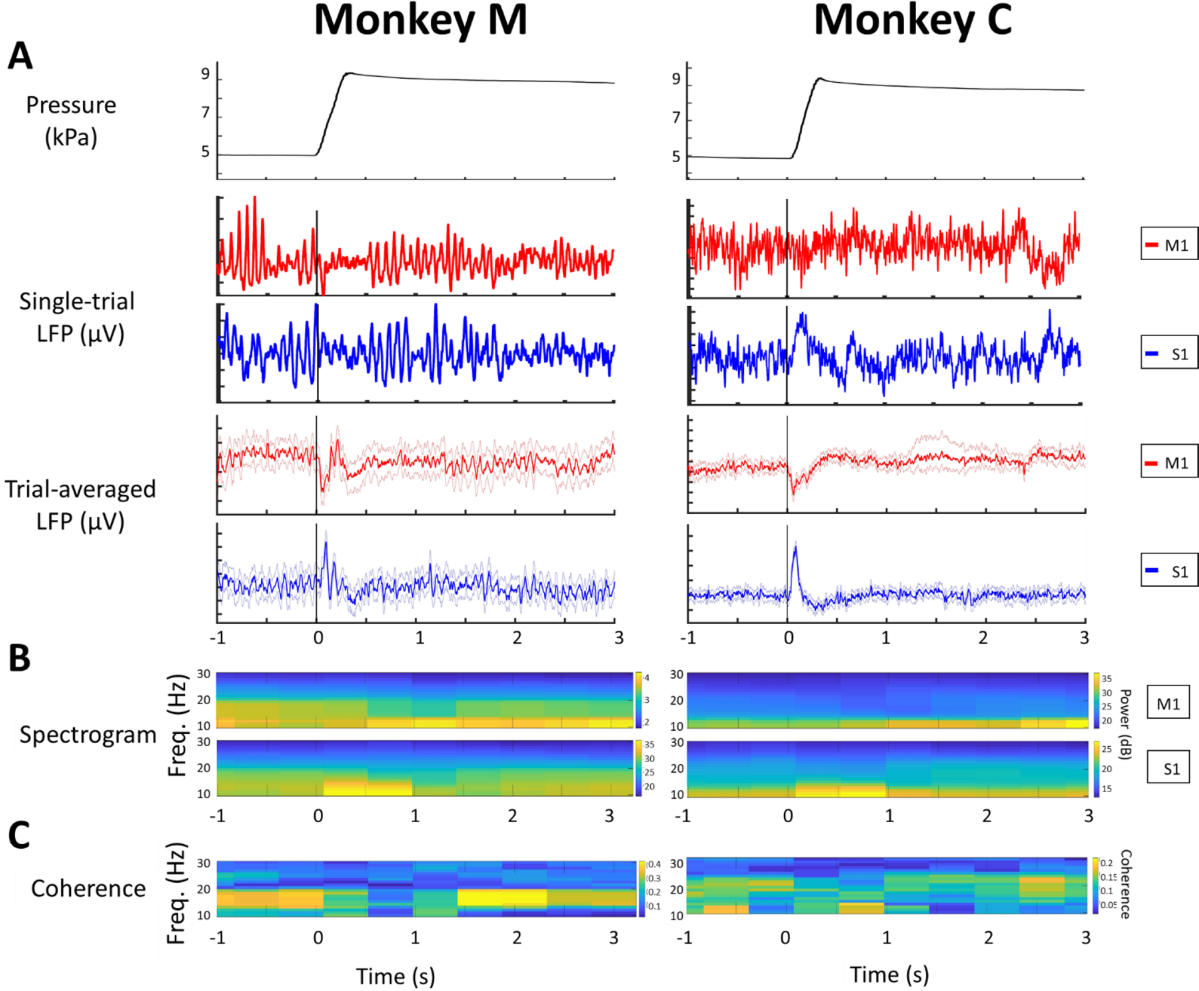
Single LFP S1–M1 pair activity when the ‘SURPRISE EVENT’ is introduced. Here all trials were aligned to the onset of the abrupt compliance change (t = 0). Left: Monkey M. Right: Monkey C. The change in compliance modulates neural signals in both time and frequency domains. Data is shown from selected channel pairs, averaged across all trials (n = 51 for monkey M and n = 86 for monkey C). (**A**) Upper panel: Time-varying internal pressure of the manipulandum from 0 to 250 ms. Middle panels: LFP signals recorded from one M1 and one S1 channel shows a pronounced reduction in beta-band activity during the SURPRISE EVENT. Well-defined beta-band activity returned once the animal entered the new steady-state. Lower panels: Trial-averaged LFP response in one channel for the S1 and M1 signals shows modulation at the t = 0 (bootstrap resampling, the number of resamples is 1000). The bold red and blue lines showed the mean of LFP amplitude across trials and the shaded regions denoted 95% confidence interval of the data (n = 51 trials collected in 3 sessions for monkey M and 86 trials collected in 3 sessions for monkey C). (**B**) Trial-averaged LFP spectrogram during the ‘SURPRISE EVENT’. Upper panel: M1 spectrogram. Lower panel: S1 spectrogram. (**C**) Spectro-temporal S1–M1 LFP coherence (shown as a heat map) likewise reflects the presence of beta coherence (Random permutation test, #permutation = 200, p = 10^–2^) during both the old and the new steady-state.

Next, we explored cortical communication during the ‘SURPRISE EVENT’, i.e. the impact from introduction of a sudden change in the compliance of the manipulandum. While the monkey was squeezing the manipulandum in steady state, the pressure in the manipulandum was increased at random time points across multiple trials by approximately 4 kPa, the reduction in compliance occurring within ~ 200 ms, (dashed line in Fig. 1B and top trace of Fig. 5A). To illustrate, the middle traces in Fig. 5A show single trial LFP traces for both S1 and M1 where the time origin, t = 0, now designates the onset of the abrupt compliance decrease.

Trial-averaged LFP (lowest trace in Fig. 5A) shows that in both S1 and M1 ERPs near t = 0, similar to the modulation seen at the onset of touch in NORMAL task (Fig. 2A). In both monkeys we documented a negative discharge of M1 signals followed by a slow recovery. By contrast, we found a strong and fast positive polarization for S1, followed by a slower depolarization. Broadly, these evoked-potentials occurred around 40–80 ms following the start of the abrupt compliance change at t = 0, lasting approximately 300–600 ms.

Analysis of the LFP spectrograms showed likewise the presence of significant evoked modulation at the onset of the imposed compliance change (Fig. 5B). We observed a near complete replacement of beta oscillations by a low-frequency, large-amplitude ERP (bootstrap resampling, the number of resamples is 1000). This spectral modulation occurred around 40-100 ms after t = 0 (the onset of the ‘SURPRISE EVENT’) and lasted up to 1 s. Thereafter, the ERP power density decreased and was replaced by the re-emergence of the robust beta-band signals once the monkey had found a new stable steady-squeeze state point. We labelled this latter stage as the “Recovery” process. The beta oscillation peak frequencies were consistent with the ones in the first steady-state (Supplementary Fig. 1B).

Single pair coherence and average coherence plotted as heat maps give insight to the dynamics of LFP interarea coherence. We found statistically significant levels of LFP coherence in the beta range prior to the onset of the SURPRISE EVENT (from −2 to 0 s), consistent with the result from the ‘NORMAL task. At t = 0, the beta coherence ramped down with the disappearance of beta frequency (spectrograms of Fig. 5C for S1 and M1). In both monkeys, this reduction in coherence lasted approximately 1 s, where after the beta-band coherence rallied, as the animal’s hand squeeze reached a new steady-state.

### Abrupt and randomly timed change in compliance interrupts bidirectional S1-M1 LFP communication, which recovers when the animal finds a new steady-state

**Figure 6:**
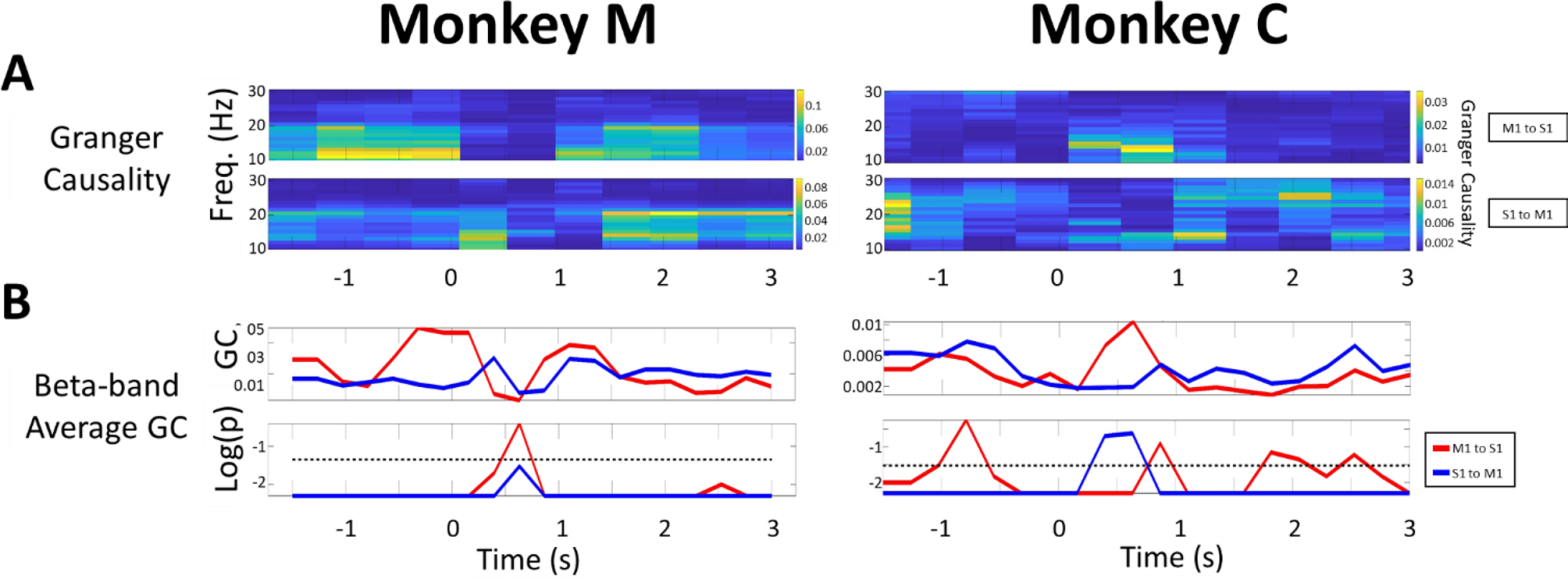
Granger Causality for a single S1–M1 LFP channel pair shows the interruption of beta-band Granger Causality from the onset of the SURPRISE EVENT at t = 0. Left: Monkey M. Right: Monkey C. Data is averaged across all trials (n = 51 for monkey M and n = 86 for monkey C). (**A**) Spectra-temporal S1–M1 LFP Granger Causality heat map indicates the bidirectional beta-band causality between S1 and M1 in both monkeys in the initial steady-state. Causality decreased significantly during the onset of the SURPRISE EVENT but recovered thereafter. Upper panel: Granger causality from M1→ S1. Lower panel: Granger causality from S1→ M1. (**B**) Upper panel: Averaged Granger causality across the beta-band (15–30 Hz) for a single LFP channel pair reflects the vanishing of the beta-band GC index at the onset of the SURPRISE EVENT. Once the animal found the new steady-state, the GC values gradually recovered. Lower panel: p-value in log_10_ scale calculated from random permutation test (# permutations = 200). We chose a target α = 0.05 and used FDR correction for multiple testing. The black dashed line represents the log of adjusted α. Those GC indices with p-value above the dashed line were considered as statistically not significant.

We again resorted to the Granger Causality in examining the impact of the compliance perturbation on S1 ↔ M1 communication. We computed the GC index while focusing specifically on a 5 s interval around the onset of the SURPRISE EVENT. We first computed the GC for a selected single LFP channel pair to find what effect, if any, did our recordings unveil. Figure 6A shows the heat map of the GC index for this S1–M1 pair. During the initial steady squeeze preceding the compliance change, i.e. the interval from −2 to 0 s, the GC values for both S1→ M1 and M1→ S1 directions showed expected levels of causation as in the NORMAL task. Following the surprise event at t = 0, the GC index in both directions decreased significantly (Fig. 6B). However, once the animal had re-calibrated his hand squeeze (force), typically in 1 s, a new steady-state was established and the GC index recovered.

**Figure 7:**
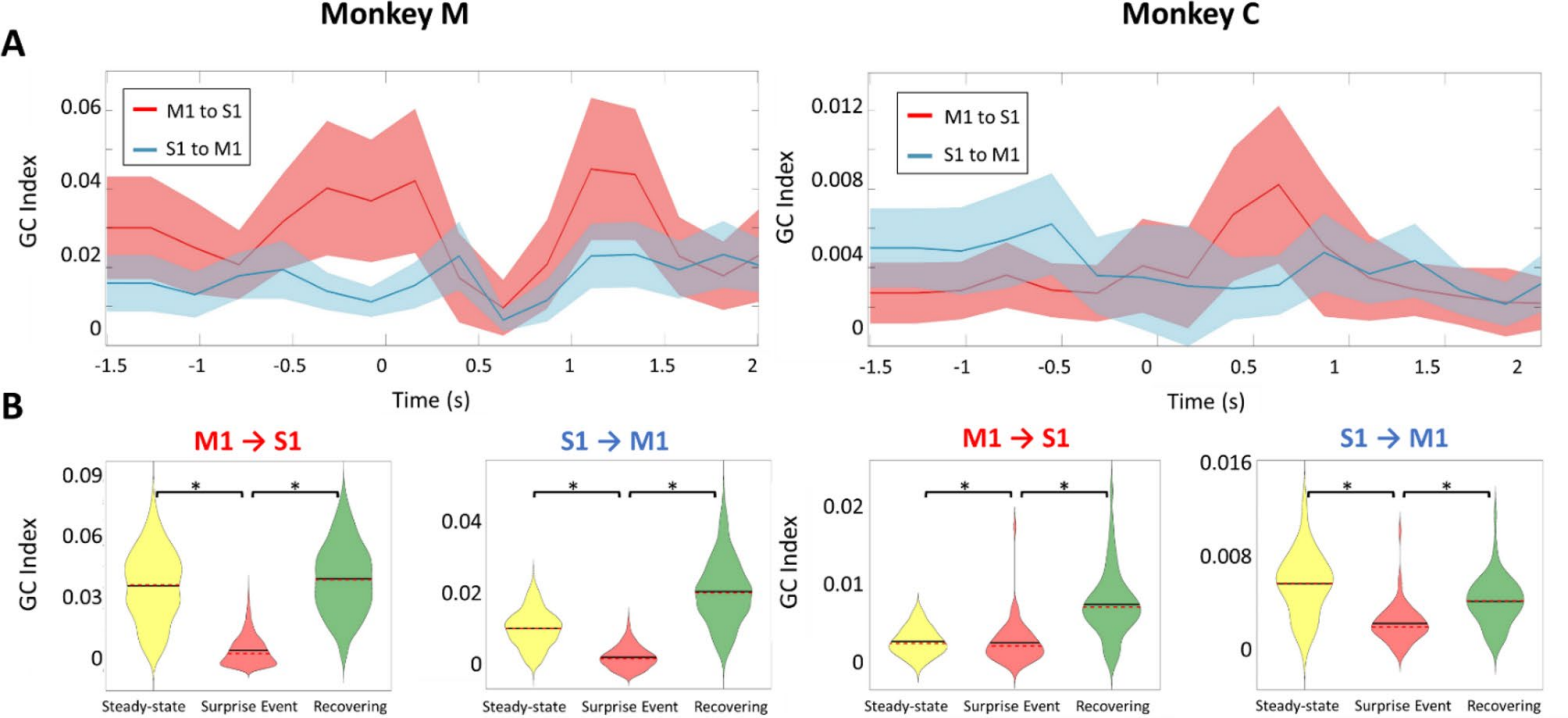
Channel-averaged Granger causality shows that S1-M1 communication channel is modulated by a sudden tactile experience (‘SURPRISE EVENT’). Left: Monkey M. Right: Monkey C. All trials were aligned on the onset of compliance change at t = 0. (**A**) Beta-band averaged, channel-averaged Granger causality index computed using a sliding window (length = 500 ms, step = 250 ms) for each direction of cortical communication. p-value calculate from random permutation test (# permutations = 200) We chose a target α = 0.05 and used FDR correction for multiple testing. M1- > S1, Red shadowed trace; S1- > M1, Blue shadowed trace. The bold red and blue lines showed the mean of GC values across LFP pairs, and the shaded regions denoted one standard deviation of the data. (**B**) Violin plots of the distribution in the GC index from the pool of 1310 selected pairs in monkey M and 898 pairs in monkey C for the three stages of each trial. The GC values in the Steady-state and Recovery stages were larger than Surprise Event stage. (*p = 10^–3^, two-sample t-test).

For statistical evaluation across multiple microelectrodes, we computed averaged GC values across many LFP S1–M1 channel pairs (1310 selected pairs in monkey M and 898 pairs in monkey C). We found overall similarity to the case of a single channel pair above. Again, the averaged GC values were statistically significant during the steady-state preceding the ‘SURPRISE EVENT’ as found in the ‘NORMAL’ task (Fig. 7A). As the compliance of the manipulandum was changed, a pronounced decrease in the statistically averaged GC indices for both S1→ M1 and M1→ S1 directions occurred. The GC index reached a minimum at approximately 0.4–0.5 s, consistent with the observed LFP envelope modulation in Fig. 5. We suggest that this loss of causality indicates partial interruption in the inter-areal communication, mirrored in the beta band activity, allowing the involved cortical areas to adapt to the unexpected perturbation for the network find a new steady state. The GC index recovered quite quickly to reach a maximum in approximately 1–1.5 s in monkey M and 0.5–1 s in monkey C.

As a statistical summary, Fig. 7B shows the ‘violin plots’ in the distribution of the GC index across the three-time intervals of interest here: the initial steady-state squeeze, the SURPRISE EVENT, and the Recovery stage leading to a new steady-state of the hand squeeze (prior to hand release). As expected, the reduction in bidirectional communication is reflected in the violin plots. Quantitatively, for Monkey C, the index for S1→ M1 is highest in the steady-state period, while M1→ S1 showed the highest (relative) values during the Recovery period. For Monkey M, the M1→ S1 index appeared to dominate the both Steady-state and Recovery period. (Two-sample t-test, p = 10^–3^ for each).

## Discussion

Dexterous manipulation of compliant objects is thought to involve the coordinated activity of both sensory and motor cortices, in particular S1 and M1. To our knowledge, this study is the first to examine directed S1 ↔ M1 interactions during these types of dynamic, soft object manipulations. Broveli et al.^12^ examined directed S1  ↔  M1 interactions based on Granger causality, but in a different context of movement preparation during visually-guided tasks. The closest to our study is the previous work by Lemon and colleagues^27^ on the (isometric force) precision grip of non-compliant objects. Further, we examined a task involving controlled yet randomly administered perturbations of object compliance (“SURPRISE EVENT”) leading to dynamic adaptation and distinct steady sensorimotor states while recording intracortical LFPs via dense microelectrode arrays placed in S1 and M1. Our results demonstrate that S1 and M1 interactions are bidirectional and dynamic, being sustained primarily during the steady squeeze of the compliant objects and attenuating during adaptations induced by the perturbations. Overall, our findings emphasize the role of transient beta oscillations in the maintenance of steady sensorimotor states involving no significant changes in movement kinematics. They also indicate that transitions between these steady motor states require transient attenuation or termination of beta activity in both S1 and M1 neuronal populations.

Presumably, there should also be communication between S1 and M1 shortly after surprise perturbation and touch events, which are correlated with attenuation of beta activity. These events are expected to lead to the arrival of related sensory information and motor updates in S1 and M1. Because of its roughly event time-locked nature, this communication tends to be accompanied by average event-related potentials which typically include both lower and higher frequency components than the examined beta transients. However, the examination and interpretation of cortico-cortical interactions detected via GC measures is much more complicated during these ERPs. That is because of artifacts introduced by the common across-trial ERP nonstationarities in these types of neural recordings and tasks^28,29^. For example, slow nonstationarities in global brain excitability (due to fatigue, fluctuations in attention, etc.) over the course of an experimental session can affect the amplitudes and latency onsets of single-trial ERPs across different brain areas in a correlated way. These correlations, even though originating from slow nonstationarities, can contribute spurious effects in the estimation of transient interactions in the much faster time scales (tens to hundreds of milliseconds) of interest here. Common approaches to address these artifacts by properly separating these different time scales’ effects involve extensive modeling and analyses, which would be better presented in a separate study. For this reason, here we have focused only on steady-state interactions reflected in transient LFP beta oscillations. Similarly, the examination of directed interactions at the level of the recorded single-unit activity^30–32^ during the different stages of this task is beyond the scope of this manuscript. We hope to address these issues in future studies.

### Differences in beta-band activity in the two subjects

In the spectro-temporal summary of Figs. 3 and 6, we found differences between the two monkeys in the frequency of their respective beta-band oscillations. We observed that (i) the frequency was notably lower in the aged Monkey M (11 years old; ~ 13 to 16 Hz) in comparison with the younger Monkey C (6 years old; ~ 20 to 30 Hz, Supplementary Fig. 1); and further (ii) that the beta-band power density in Monkey M was higher than in Monkey C in both M1 area and S1 area. Nevertheless, several commonalities between the two animals were statistically clear: (i) The overall time course of the temporal GC patterns for the NORMAL task and SURPRISE EVENT Task was very similar; (ii) as for the direction of cortical communication, the GC for Monkey M (M1 → S1) was mostly driven by M1 and the same dominance showed in Monkey C although the “gap” between M1→ S1 and S1–M1 was smaller; (iii) The spectral GC maps showed similar results as did (iv) the spectral field coherence. We also note that difference in power in given frequency bands across the two subjects could be due to differences in impedance and other related properties of the implanted microelectrode arrays.

There is literature about the variation in beta band characteristics as a function of age. Studies in healthy humans have attributed the lower beta-band peak frequency^26^ in older subjects to the greater GABA-inhibition in sensorimotor cortices. Elsewhere, Hubner and colleagues^25^ measured beta oscillations during a grip-force modulation task of rigid manipulanda and showed aging affecting the beta power (older subjects exhibit decrement of beta task-related power). Studies focusing on cortical activity in the resting stage have shown that older adults demonstrated higher beta-band power comparing to young adults^33^. Bercovitch and collaborators^34^ conducted a longitudinal study of macaques related to body conditions and reproductive health which, while quite indirect, could be viewed as connected with the slower beta band in an old animal. We suggest that such age factors could be also the reason for the quantitative differences in the magnitude of the Granger Causality index for our two monkeys. Beyond the possible age-dependent factor, the difference in LFP signals across animals could arise from other uncontrolled factors such as the relative placements of the microelectrode arrays, the exact depth of their implantation, or individual differences in the animals’ individual traits.

### Granger causality reflects bi-directional communication during steady state hand squeeze

Modeling of inter-area cortical communication has included the role of coherence^11^, time causal interactions^12,19^ and communication via cortical subspaces^35^. There have been several studies on neural dynamics associated with hand/arm reach and grasp of non-compliant (rigid) objects using spikes and/or field potentials recorded from the motor cortex alone^36^. Connecting with previous studies^37,38^ we also observed how the LFP beta power ramped down during the initial arm/hand kinematics as the monkeys reached for the manipulandum.

The stage of the task of main interest to us was the relatively long (3–4 s) period when the animal squeezed the compliant manipulandum with a specific, instructed isometric force. We reasoned that a bidirectional S1 ↔ M1 communication channel in such a steady state would be active, on the assumption that the monkey uses tactile input to guide its motor output and vice versa. Following the initial increase of the GC index after the onset of touch, similar to study by Reyes et al.^39^, the data shows clearly how a maximum value is reached once the animal found the required stable point of squeeze, now in steady-state, supporting the hypothesis of enhanced cortico-cortical communication.

### Granger causality is modulated by an unexpected change in tactile feedback

In the SURPRISE EVENT, the animal had to alter the force of the grip to squeeze the manipulandum to reach a new target pressure level. From the neural data it appears that the monkeys were briefly confounded by the change of compliance but quickly searched for and found the grasp condition appropriate for a new steady state. As pointed out in Figs. 5A,B, we found that during the approximately 0.3 s duration of the SURPRISE EVENT the LFP signals exhibited ERPs in both S1 and M1, presumably due modulation of information transmitted from the cutaneous hand receptors to the cortex^40^. In parallel, the beta-band oscillations reduced significantly, qualitatively similar to their damping at the first onset of touch (Fig. 2). Interestingly, the time domain GC index values in both directions (S1 → M1 and M1 → S1) decreased significantly during the transient event of the compliance change until approximately 0.5 s beyond the event. We speculate that such transient downmodulation in the S1-M1 communication might be due to the timing in the arrival of thalamic inputs to S1 and a corresponding adaptation by M1 to allow for changes e.g., in maintenance of posture during the re-calibration of squeeze force. A temporary interruption in cortico-cortical communication in the beta frequency might shift the initial balance between M1 and S1 whereby the latter helps drive the cortex towards a new balance between the two networks.

### Role of beta activity in sensorimotor networks

While cortico-cortical communication has been reported to involve multiple frequency bands^14,27,41^, the importance of beta oscillations in sensorimotor function has been broadly debated in the literature including the so-called “status quo” argument for maintaining and signaling the status-quo of sensorimotor and cognitive processes^42^. In addition, in the context of cortical and basal ganglia networks, it has been proposed that transient beta oscillations may reflect a likelihood index for the need of voluntary interventions to change sensorimotor states^43^. However, the precise function of beta-synchrony remains open^44^. More recently, Bastos et al.^14^ have presented supporting evidence for beta activity involvement on top-down organization of bottom-up sensory streams (mostly involving gamma oscillations) in the context of attentional effects or more broadly in terms of information flow in brain neural networks.

To be sure, transient beta oscillations might have diverse biophysical mechanisms and support many of the non-exclusive roles cited above. Our findings in the specific context of dynamic maintenance of steady grasping states of compliant objects support the status-quo maintenance role. However, our preference is to state this in a more specific fashion in terms of steady-state maintenance of certain kinds of steady sensorimotor states. Specifically, those steady-states where kinematics, in particular position configurations of limbs, hands, fingers, etc., do not change significantly during periods of time. It remains to be shown whether other types of steady-states, e.g. steady ongoing rhythmic locomotion states without requiring volitional intervention, might also involve beta oscillations.

## Supplementary Information

Supplementary Figures.

## Data Availability

All data generated during the current study are available from the corresponding author upon reasonable request.
