# Supplementary material for: Grasp-squeeze adaptation to changes in object compliance leads to dynamic beta-band communication between primary somatosensory and motor cortices
Source: Sci Rep. 2022 Apr 26;12:6776. doi: 10.1038/s41598-022-10871-z (PMC9042850; doi:10.1038/s41598-022-10871-z)
Supplement: Supplementary file 1 — Supplementary Figures. [file 41598_2022_10871_MOESM1_ESM.pdf]

GRASP-SQUEEZE ADAPTATION TO CHANGES IN OBJECT COMPLIANCE LEADS TO DYNAMIC BETA-BAND COMMUNICATION BETWEEN PRIMARY SOMATOSENSORY AND MOTOR CORTICES

HUY CU[1] *, LAURIE LYNCH[2], KEVIN HUANG[4], WILSON TRUCCOLO[2,3], ARTO NURMIKKO[1,3] *

[1]School of Engineering, Brown University, Providence, RI, United States.
[2]Department of Neuroscience, Brown University, Providence, RI, United States.
[3]Carney Institute for Brain Science, Brown University, Providence, RI, United States.
[4]Department of Neurosurgery, Brigham and Women's Hospital, Harvard Medical School, Boston, MA, United States.

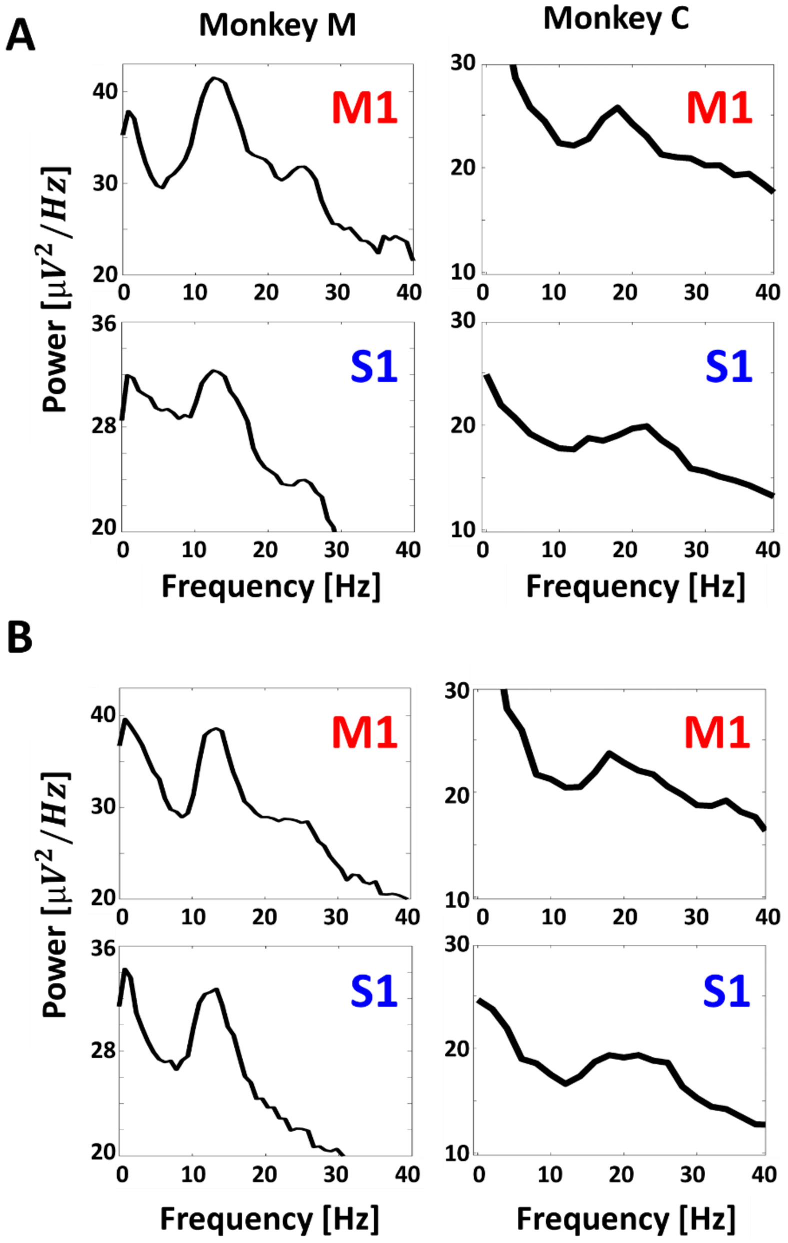

Supplemental Figure 1. *Power spectra of the M1 and S1 LFP recordings in both monkeys during (**A**) the Steady-state and (**B**) the Recovery stage. The power spectral density was estimated using multi-taper method with 7 tapers within a 1-second window length (n = 51 trials collected in 3 sessions for monkey M and 86 trials collected in 3 sessions for monkey C). In monkey M (left), the beta peak locates approximately at 13.5Hz in both S1 and M1. In monkey C (right), the beta peak occurs at 19Hz in M1 and 23Hz in S1. All trials were aligned on the onsets of SURPRISE EVENT (t=0).*

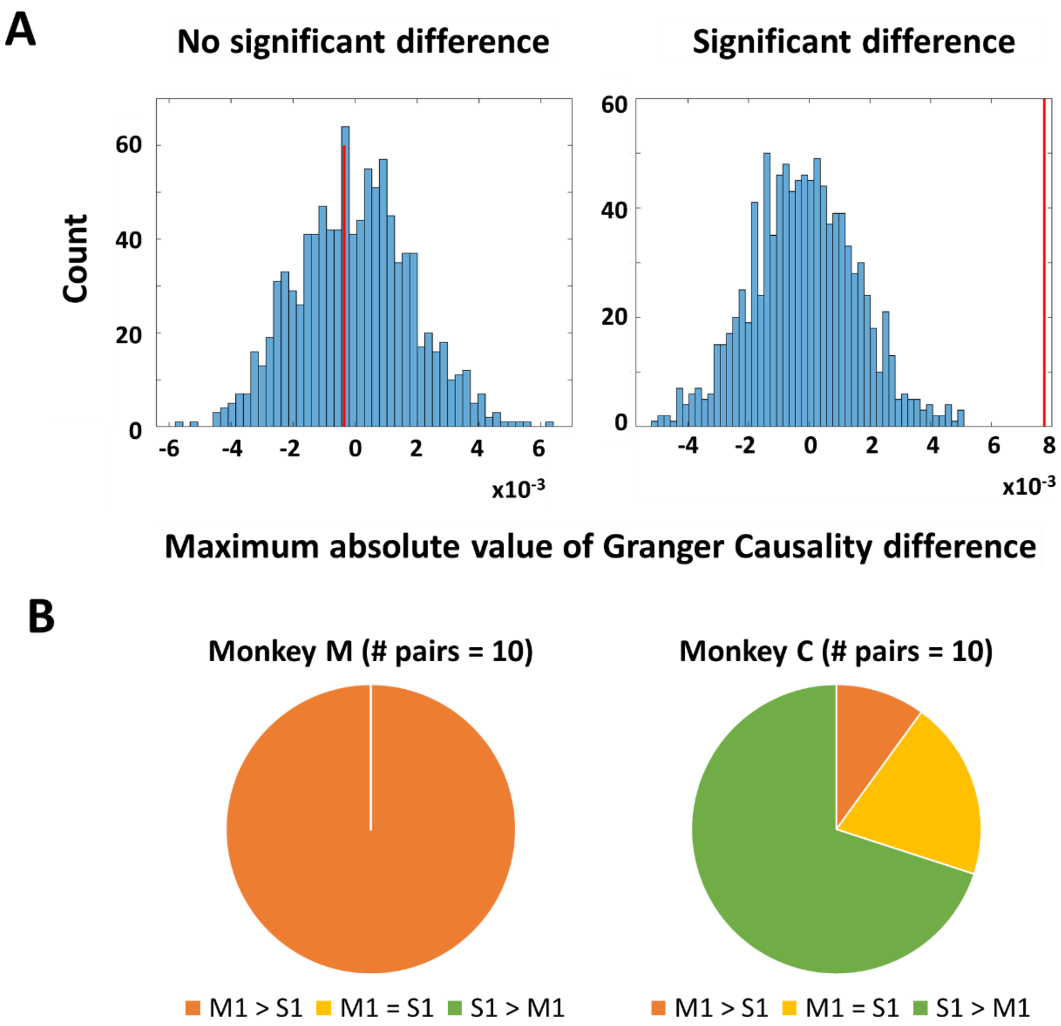

Supplemental Figure 2*. Random permutation test (# permutations = 1000) during the Steady-state for the monkeys. We chose a target α =0.05 and used FDR correction for multiple testing. (****A****) Distribution of values in the difference between M1->S1 and S1->M1 GC values under the null hypothesis of no difference between the two conditions (M1-> S1 and S1->M1). Red line indicates the real difference from two GC measures in the real data set. (****B****) During the Steady-state, 10/10 LFP pairs of monkey M showed that M1->S1 is the leading direction. Meanwhile, in monkey C, 7/10 pairs showed that S1-->M1 is the main direction, 1/10 pair supported M1->S1 direction and 2/10 pairs supports the null hypothesis.*
